# Similar pharmacokinetics and pharmacodynamics of a new biosimilar and reference insulin aspart in healthy Chinese males

**DOI:** 10.1038/s41598-021-88782-8

**Published:** 2021-05-04

**Authors:** Hui Liu, Hongling Yu, Lisi Sun, Jingtao Qiao, Sainan Wan, Shuang Li, Jiaqi Li, Huiwen Tan, Yerong Yu

**Affiliations:** 1 Department of Endocrinology and Metabolism, West China Hospital, Sichuan University, No.37 Guoxue Alley, Wuhou District, Chengdu City, Sichuan Province People’s Republic of China; 2 Yichang HEC Changjiang Pharmaceutical Co., Ltd., No. 38 Binjiang Road, Yidu, Yichang City, Hubei Province People’s Republic of China

**Keywords:** Drug discovery, Endocrinology

## Abstract

Insulin aspart (IAsp) is one of the main therapies used to control blood glucose after a meal. This study aimed to compare the pharmacokinetics (PK) and pharmacodynamics (PD) of 2 rapid-acting IAsp products: a new IAsp biosimilar (RD10046) and NovoRapid. In a single-center, randomized, single-dose, 2-period, crossover, euglycemic clamp study (registry number: CTR20180517, registration date: 2018-05-30), healthy Chinese males were randomized to receive 0.2 U/kg of the IAsp biosimilar RD10046 and NovoRapid under fasted conditions on two separate occasions. PK and PD were assessed for up to 10 h. Of the 30 randomized subjects, all 30 completed both treatment periods. The PK (area under the curve [AUC] of total IAsp; maximum observed IAsp concentration [C_max_]) and PD (maximum glucose infusion rate [GIR_max_]; total glucose infusion during the clamp [AUC_GIR,0–10h_]) were similar between the new IAsp biosimilar RD10046 and NovoRapid. In all cases, the 90% CIs for the ratios of the geometric means were completely contained in the prespecified acceptance limits of 0.80–1.25. No hypoglycemic events, allergic reactions, or local injection adverse reactions occurred in this trial. We concluded that the studied IAsp biosimilar (RD10046) was bioequivalent to NovoRapid.

## Introduction

Insulin is one of the main therapies used to treat diabetes mellitus. Insulin aspart (IAsp), a kind of rapid-acting insulin in which the amino acid proline in the b-chain has been replaced by the amino acid aspartic acid, is manufactured using recombinant DNA technology^1,2^. The time of onset of action of IAsp is faster, and the duration of action is shorter than that of human insulin despite being equally potent in terms of glucose-lowering effects^3–5^, which leads to greater reductions in postprandial glucose excursions.

RD10046 is a non-innovator recombinant insulin aspart formulation developed by Yichang HEC Changjiang Pharmaceutical Co., Ltd. (HEC Pharm). It was developed in accordance with biosimilar guidelines established by the European Medicines Agency (EMA)^6^, the US Food and Drug Administration^7,8^, and the China Food and Drug Administration^9^. These guidelines recommend a pharmacokinetic (PK) and pharmacodynamic (PD) comparison of a new insulin analog with a reference insulin in glucose clamp studies. This article presents the results of a single-center, randomized, single-dose, 2-period, crossover, euglycemic clamp study in healthy males to evaluate similarities in the pharmacokinetic and pharmacodynamic properties of RD10046 and NovoRapid (Novo Nordisk, Denmark). The main objectives of the study were to (1) demonstrate average bioequivalence (BE) in the PK/PD endpoints between RD10046 and NovoRapid and (2) assess the safety of the two insulin preparations.

## Results

### Subjects disposition, demographics

**Figure 1:**
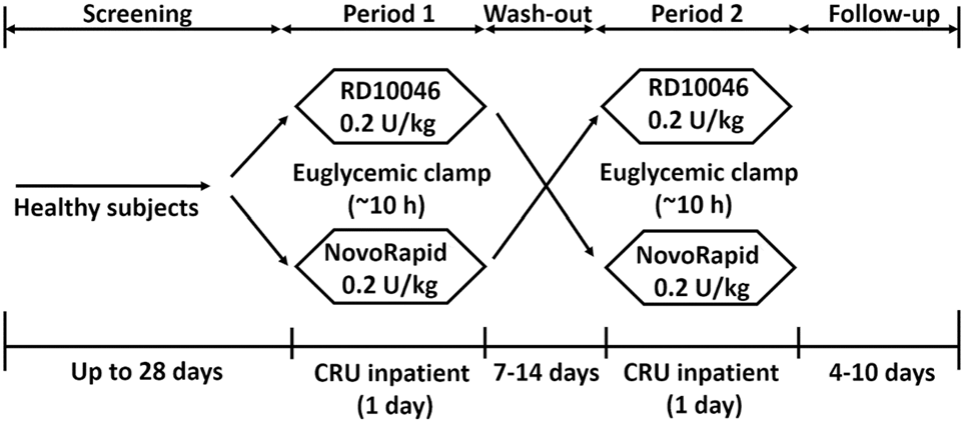
Study design. *CRU*, clinical research unit.

In total, 30 healthy male subjects were enrolled after eligibility evaluation; of these, 30 subjects were randomized in a 1:1 ratio to the two treatment arms (sequences) (Fig. 1). The subjects were between 21 and 33 years old with a mean value of 24.7 years old. Subject height, weight, and BMI ranged from 161.5 to 181.0 cm, 52.7 to 77.0 kg, and 19.1 to 24.0 kg/m^2^, respectively, with mean values of 171.5 cm, 64.3 kg, and 21.8 kg/m^2^ at the screening visit, respectively. The weight and BMI on the day of dosing were shown in Table 1.

**Table 1:** Demographics of subjects and index to estimate the quality of euglycemic clamps.

	RD10046 N = 30	NovoRapid N = 30	*P*
**Demographics of subjects**			
Age (year)^a^	24.7 ± 2.89	24.7 ± 2.89	–
Weight (kg)^a^	63.5 ± 5.87	63.6 ± 5.84	0.97
BMI (kg/m^2^)^a^	21.6 ± 1.40	21.6 ± 1.43	0.95
**Quality control index**			
Target of BG (mmol/L)^a^	4.33 ± 0.31	4.35 ± 0.27	0.93
‘Clamped’ BG (mmol/L)^a^	4.36 ± 0.26	4.34 ± 0.23	0.70
Basal C-peptide (pmol/L)^a^	300 ± 83.7	288 ± 71.0	0.52
Postdosing C-peptide (pmol/L)^a^	206 ± 63.3	200 ± 63.0	0.64
C-peptide suppression after injection (%)^a^	30.2 ± 14.7	30.7 ± 13.3	0.89
CVBG (%)^a^	4.13 ± 0.99	4.03 ± 0.82	0.68
MARD at target BG level (%)^a,b^	3.56 ± 1.00	3.38 ± 0.75	0.43

*BMI* body mass index, *CVBG* coefficient of variation of blood glucose, *BG* blood glucose, *MARD* mean absolute relative difference.

^a^Mean ± SD.

^b^MARD at the target BG level (%): 100 ×|(clamped BG − target BG)/target BG|.

### Euglycemic clamp statistics and C-peptide levels

**Figure 2:**
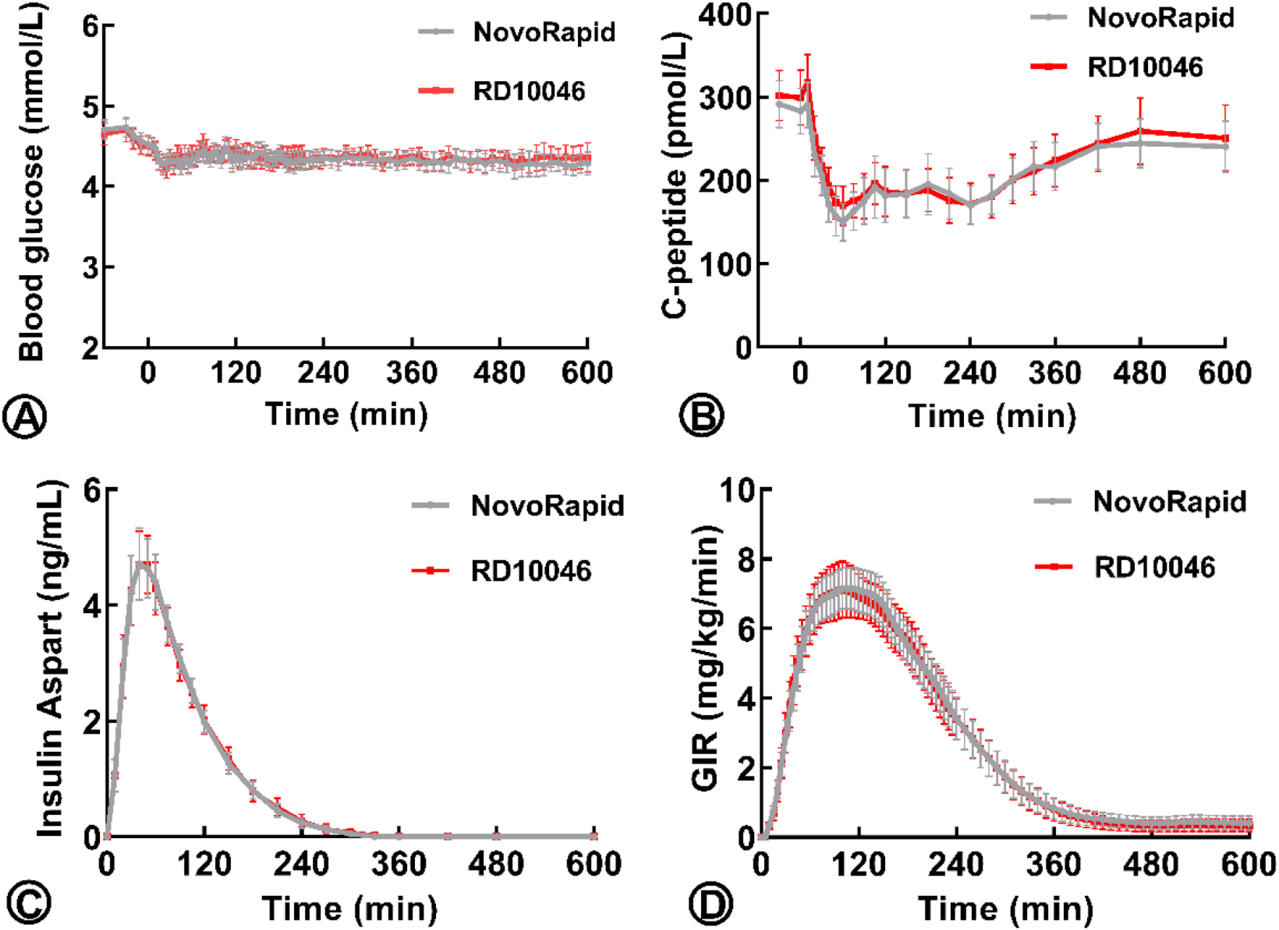
The mean value of clamped blood glucose (**A**), C-peptide (**B**), insulin aspart level (**C**), and glucose infusion rate (GIR) (**D**) in the RD10046 and NovoRapid group during the euglycemic clamps. The error bars represent the 95% confidence intervals.

The fasting baseline blood glucose (BG) for each euglycemic clamp was defined as the mean value of several glucose measurements (− 60, − 30, − 20, − 10 min) before IAsp administration. Following drug administration, the onset of insulin action was defined as the time when BG dropped below the target level, which was defined as 5 mg/dL (0.28 mmol/L) less than the subject’s fasting baseline. The baseline BG levels were 4.61 ± 0.31 mmol/L in the RD10046 group and 4.63 ± 0.27 mmol/L in the NovoRapid group (*P* = 0.93). The target BG levels were 4.33 ± 0.31 and 4.35 ± 0.27 mmol/L in the two groups, respectively. As shown in Fig. 2A, the clamped BGs were comparable (4.36 ± 0.26 mmol/L in the RD10046 group and 4.34 ± 0.23 mmol/L in the NovoRapid group, *P* = 0.70). The ‘clamped’ BGs were also close to their own target levels [4.36 ± 0.26 *vs.* 4.33 ± 0.31 mmol/L in the RD10046 group (*P* = 0.69) and 4.34 ± 0.23 *vs.* 4.35 ± 0.27 mmol/L in the NovoRapid group (*P* = 0.88)]. The coefficient of variation in BG (CVBG) was relatively low and comparable in both groups (4.13% ± 0.99% and 4.03% ± 0.82% in the RD10046 group and NovoRapid group, respectively, *P* = 0.68). The mean absolute relative difference (MARD) at the target BG level was 3.56% ± 1.00% and 3.38% ± 0.75% for the two groups, respectively (*P* = 0.43).

As shown in Fig. 2B and Table 1, the mean values of basal C-peptide were 300 and 288 pmol/L for the RD10046 and NovoRapid groups, respectively. The overall C-peptide levels after dosing were 206 ± 63.3 and 200 ± 63.0 pmol/L, respectively. A mean reduction of 30.2% and 30.7% from baseline was observed, respectively. The AUC_0–10h_ of C-peptide were 139 ± 11.8 and 135 ± 9.77 nmol/L × min in groups RD10046 and NovoRapid respectively (*P* = 0.16). The mean C-peptide concentration profiles after RD10046 and NovoRapid subcutaneous injection were similar, indicating comparable (Fig. 2B) endogenous insulin production between the two treatments. For this reason, the interference of endogenous insulin to the observed GIR was speculated to be equal between the two treatments. Only one experiment in the RD10046 group showed significant increases in postdosing C-peptide from baseline (e.g., > 200 pmol/L)^10^, and as a result, this experiment was excluded from PD analysis.

### Pharmacokinetics

The subjects received a subcutaneous injection of RD10046 or NovoRapid (0.2 U/kg) on two separate occasions with an interval of 7–14 days. Subjects were weighed on the morning of the experiment day under fasting conditions. The doses of IAsp were 12.7 ± 1.1 U for the RD10046 group and 12.7 ± 1.2 U for the NovoRapid group (*P* = 0.91).

The mean insulin concentration-versus-time profiles were similar between formulations (Table 2, Fig. 2C). The similarity in PK profiles was confirmed by the ratio of least squares (LS) means for AUC_IAsp,0–10h,_ and C_max_ values. Moreover, the equivalent PK of RD10046 relative to NovoRapid was demonstrated by the 90% CIs for the exposure ratios within 0.80–1.25. The time to the observed maximum drug concentration (T_max_) was almost the same between formulations, with a median difference of 0 min and a 95% CI of the difference from − 10 to 10 min.

**Table 2:** Parameters of pharmacokinetics and pharmacodynamics.

	RD10046	NovoRapid	Ratio^a^ or difference^b^ of LS means	90% CI (ratio^a^), 95% CI (difference^b^)
**PK parameters**				
N	30	30	**–**	**–**
C_max_ (ng/mL)	4.84 (31) 5.08^c^ (1.55)^d^	4.78 (31) 5.00^c^ (1.55)^d^	1.01^a^	(0.93,1.11)^a^
T_max_ (min)^e^	50.3 (20.4,60.9)	50.2 (30.2,75.3)	0.00^b^	(− 10,10)^b^
t_1/2_ (min)^c,d^	39.2 (13.1)	38.0 (9.38)	–	–
AUC_IAsp,0–10h_ (ng/mL × min)	514 (14.6) 503^c^ (73.5)^d^	504 (17.0) 495^c^ (84.0)^d^	1.02^a^	(0.99,1.06)^a^
AUC_IAsp,0–∞_ (ng/mL × min)	514 (13.9) 519^c^ (72.0)^d^	504 (16.5) 510^c^ (84.0)^d^	1.02	(0.99,1.06)^a^
**PD parameters**				
N	29	30	–	–
GIR_max_ (mg/kg/min)	7.44 (23) 7.65^c^ (1.74)^d^	7.64 (22) 7.84^c^ (1.72)^d^	0.98^a^	(0.92,1.05)^a^
tGIR_max_ (min)	113 (33)	104 (26)	15^b^	(− 10,30)^b^
T_early,50%_ (min)	38 (21)	38 (22)	0.13^b^	(− 3.65,4.03)^b^
T_late,50%_ (min)	227 (21)	226 (17)	0.39^b^	(− 24.13,25.39)^b^
T_onset_ (min)	16 (28)	15 (23)	0^b^	(0,5)^b^
AUC_GIR,0–10h_ (mg/kg)	1493 (31) 1558^c^ (478)^d^	1542 (27) 1594^c^ (427)^d^	0.97^a^	(0.91,1.04)^a^

Values are presented as geometric means and CV (%) unless otherwise indicated.

*AUC*_*0–10h*_ area under the curve from time 0 to 10 h, *AUC*_*0–∞*_ area under the curve from zero to infinity, *CI* confidence interval, *C*_*max*_ maximum observed drug concentration, *CV* coefficient of variation, *GIR* glucose infusion rate, *h* hours, *LS* least squares, *N* number of subjects, *GIR*_*max*_ maximum glucose infusion rate, *T*_*max*_ time of maximum observed drug concentration, *tGIR*_*max*_ time of maximum glucose infusion rate, *T*_*early,50%*_* and T*_*late,50%*_ time of half-maximum GIR before and after GIR_max_, *T*_*onset*_ first time of the blood glucose declining down to the target level (0.28 mmol/L less than the baseline).

^a^Ratio of LS means (RD10046 divided by NovoRapid).

^b^Difference between medians (RD10046—NovoRapid).

^c^Arithmetic mean.

^d^Standard deviation.

^e^Median (range).

### Pharmacodynamics

The glucose infusion rate (GIR) was adjusted in accordance with the glucose measurement (every 5 min from 0 to 4 h and every 10 min from 4 to 10 h) to maintain the BG at the target level after dosing. The GIR reflected the glucose-lowering effect of IAsp. The GIR profiles (mean and 95% CIs) after 0.2 U/kg IAsp injection were comparable between RD10046 and NovoRapid (Fig. 2D). No difference was detected in onset time, maximum glucose infusion rate (GIR_max_), time to GIR_max_ (tGIR_max_), the area under the curve (AUC) of GIR from 0 to 10 h (AUC_GIR,0–10h_), the time for GIR to rise from 0 to half of GIR_max_ (T_early50%_), and the time for GIR to decline from GIR_max_ to half of GIR_max_ (T_late50%_) (Table 2). Median differences for PD time parameters were small (less than 15 min), and all 95% CIs encompassed zero. The 90% CI of the geometric mean ratios for AUC_GIR,0–10h_ and GIR_max_ were also entirely contained within the bioequivalent interval of 80–125%.

### Safety evaluation

Both IAsp formulations were well tolerated, and no injection-site reactions were identified. No clinically significant alterations in laboratory values, urinalysis values, or vital signs were identified. No subjects discontinued the study because of an adverse event (AE). A total of 7 mild AEs, all unlikely to be related to the study drug, were reported in 7 subjects and were unevenly distributed between the two treatments (6 in subjects treated with RD10046 *vs.* 1 in those treated with NovoRapid) (Table 3).

**Table 3:** Treatment-emergent adverse events for all causalities.

TEAE (MedDRA preferred term)	Number of AEs (number of subjects with AEs)	
	RD10046 (N = 30)	NovoRapid (N = 30)
All	6 (6)	1 (1)
Electrocardiogram high voltage	1 (1)	–
Refraction disorder	1 (1)	–
Hyperuricemia	1 (1)	–
Pain in extremity	1 (1)	–
Induration	1 (1)	–
Platelet count increased	1 (1)	–
Blood bilirubin increased	–	1 (1)

MedDRA version 21.1.

*N* number of subjects studied, *TEAE* treatment-emergent adverse events, *AE* adverse event.

## Discussion

The hyperinsulin euglycemic clamp is regarded as the gold standard for insulin sensitivity evaluation^11,12^. Further, it is widely accepted and used to investigate the PK/PD of insulin preparations^13–15^. The quality of the euglycemic clamp is of great importance because it is related to the accuracy of the assessment of PK/PD^16^. In this trial, the overall CVBG was less than 5%, and the MARD was relatively low. The clamped BG levels were also close to their target glucose (4.36 ± 0.26 *vs. *4.33 ± 0.31 mmol/L for RD10046 and 4.34 ± 0.23 *vs.* 4.35 ± 0.27 mmol/L for NovoRapid). These findings are indicative of the successful performance of the euglycemic clamp technique with blood glucose control close to the clamp target throughout the study. Furthermore, comparable baselines of blood glucose and C-peptide levels and a crossover study allowed a valid foundation for comparison of RD10046 and NovoRapid.

According to the EMA guideline^6^, either healthy subjects or type 1 diabetes patients could be used to evaluate the PK/PD bioequivalence of the two formulations. Healthy volunteers usually exhibit lower intraindividual variability than patients with type 1 diabetes mellitus, while the presence of type 1 diabetes mellitus could ensure the absence of interference of endogenous insulin. Studies in patients with type 1 diabetes would require standardized and well-controlled conditions (e.g., a long fasting period, washout of current insulin, absence of basal insulin, administration of a fixed-dose, and a run-in period before the euglycemic clamp)^17^. A study conducted in healthy subjects could reduce the pharmacokinetic variability not related to the differences between pharmaceutical products by avoiding underlying disease or concomitant medication. Thus, the present study was conducted in healthy participants. The frequently used insulin doses in clamp studies are 0.2–0.3 U/kg bodyweight for rapid-/short-acting insulins^6^. A recent study reported the PK/PD of a rapid-acting analog^18^, and the experimental procedure was similar to that in our study. The results showed that the degree of C-peptide suppression had little relationship with the injection dose of exogenous insulin (7 U, 15 U, 30 U). In the present study, the average IAsp injection dose was approximately 12 U, and the suppression of endogenous insulin secretion was approximately 30%. Although a C-peptide suppression of over 50% would have been more powerful for indicating freedom from the interference of endogenous insulin, the bioequivalence of PK/PD could still be thoroughly evaluated as long as the C-peptide concentrations were comparable between the two formulations in all periods without a C-peptide suppression of more than 50%^19^.

An overall maximum observed drug concentration appearing at approximately 50 min accompanied by a maximum GIR of 7.64 mg/kg/min and an overall glucose-lowering effect of 1542 mg/kg after 0.2 U/kg NovoRapid administration during a 10-h euglycemic clamp were observed in the present study, which was consistent with previous reports with a T_max_ of 48–70 min^3,20,21^, a GIR_max_ of 6–9 mg/kg/min^22–25^, and a total glucose infusion of approximately 1300–1500 mg/kg^23,24^. The maximum observed drug concentration and total drug exposure were higher than those in the previous report, which might be due mainly to different analysis methods. All the PK/PD parameters were similar between the new IAsp biosimilar RD10046 and NovoRapid, and the 90% CIs for the ratios of the geometric means were completely contained in the prespecified acceptance limits of 0.80–1.25. These results indicated that RD10046 was bioequivalent to NovoRapid.

The evaluation of the safety of the two formulations in this study indicated no safety concerns. A different safety profile for biosimilar insulin may arise because of a different molecular entity, a change in the structure, or different impurity profiles in the manufacturing process. Although the frequency of AEs was not equal between the two treatments, no clinically significant alterations in vital signs or laboratory results were reported, and none of the AEs were likely to be related to the two formulations.

## Conclusion

In summary, the study demonstrated that the PK (AUC_IAsp,0–10h_ and C_max_) and PD (GIR_max_ and AUC_GIR,0–10h_) properties of the biosimilar IAsp and NovoRapid were similar after single 0.2 units/kg s.c. doses in healthy Chinese subjects.

## Subject and method

### Subject

This research started on 27 Jun 2018 and ended on 25 Oct 2018. A total of 36 Chinese males were recruited and finally according to the inclusion criteria, 30 subjects were included in this clinical trial. Subjects were required to be healthy Chinese men, aged 18–45 years, with a BMI between 19.0 and 24.0 kg/m^2^, a fasting glucose level less than 6.1 mmol/L, and normal glucose tolerance. They were also required to be nonsmokers and without a family history of diabetes mellitus or hypertension and no abnormalities were found in ECG, routine blood and urine, or liver and renal function.

Trial procedures were carried out in accordance with the Declaration of Helsinki and the principles of Good Clinical Practice. All participants gave informed consent, and the study was approved by the ethics committee of West China Hospital of Sichuan University. The trial’s information was available on the website http://www.chinadrugtrials.org.cn and the registration number of this trial was CTR20180517.

### Study design

There was a phase I, single-site, randomized, single-dose, two-period, crossover, euglycemic clamp study, evaluating the similarity in PK and PD of two IAsp products in healthy subjects (a new IAsp biosimilar RD10046 by HEC Pharm vs. NovoRapid by Novo Nordisk). In this study, subjects were randomly allocated to one of two treatment sequences (Fig. 1), in which they received 0.2 units/kg s.c. doses of two different IAsp products (as listed above) on two occasions.

Subjects were admitted to the phrase I unit on day-1 of each treatment period. On day 1, subjects received a single dose of the IAsp biosimilar RD10046 or NovoRapid after an overnight fast of at least 10 h, and a euglycemic clamp procedure that lasted up to 10 h postdose was performed. Subjects were discharged when they had had carbohydrates at the end of the euglycemic clamp. An interval of 7–14 days existed between the two doses.

### Clamp procedure

The euglycemic clamp study was performed after inserting two catheters (an antecubital venous catheter for infusing 20% dextrose and a retrograde venous catheter in the hand heated in a box at 55–65℃ for measuring blood glucose). After collecting the basal blood glucose level [defined as the mean of the glucose measurement of several times (− 60, − 30, − 20, − 10 min) before dosing], the subjects received a 0.2 U/kg dose of NovoRapid or RD10046 by s.c. injection into a lifted abdominal skin-fold. Blood samples were obtained at the bedside for immediate determination of whole blood glucose concentrations and then monitored every 5 min from 0 to 240 min and every 10 min from 240 to 600 min after dosing. During the euglycemic clamp, the GIR was varied as necessary to maintain the blood glucose concentration within approximately ± 10% of the target level (defined as 0.28 mmol/L below the subject’s basal glucose level). The investigator made the GIR adjustment based on glucose measurements and experience. If the GIR fell to zero for at least 30 min, the clamp was discontinued. A 4-mL blood sample was collected at the following points for analysis of C-peptide and IAsp concentration: − 30, 0 (before dosing), 10, 20, 30, 40, 50, 60, 90, 120, 150, 180, 210, 240, 270, 300, 360, 420, 480 and 600 min. The baseline of C-peptide was defined as the mean value of two measurements at − 30 and 0 min.

### Analytical techniques

Whole blood glucose concentrations were tested with a glucose analyzer (Biosen C_line GP+, Neckar Healthcare. Co. Ltd., Magdeburg, Germany) using an automated glucose oxidase technique. Serum C-peptide levels were analyzed using an ELISA (Cat. No. 80‐CPTHU‐E01.1; ALPCO, Salem, NH), and plasma IAsp concentrations were assessed by means of a validated, ultra-performance liquid chromatography-tandem mass spectrometry (UPLC-MS/MS) method at Covance Laboratories in Shanghai. The ranges of quantification were 20–3000 pmol/L for C-peptide and 0.2–10 ng/mL for IAsp.

### Statistical methods

The primary endpoint parameters of PK were shown as follows derived from time-profiles of IAsp: (1) maximal concentration of IAsp (C_max_), (2) the AUC of IAsp from 0 to 10 h (AUC_IAsp_, _0-10h_). The time of C_max_ (T_max_) and terminal half-life (t_1/2_) were also recorded. Those parameters mentioned above were calculated by PKsolver (version 2.0)^26^. Log-transformed AUC_IAsp,0–10h_ and C_max_ were evaluated with a linear mixed-effects model including subject as a random effect with the period, sequence, and treatment as fixed effects. For each parameter, the difference in the LS means along with the 90% CIs was back-transformed to produce the ratio of geometric means and the CI comparing treatments. The pharmacokinetic similarity was to be concluded if the 90% CIs for both AUC_IAsp,0–10h_ and C_max_ were completely contained within the interval of 0.80–1.25.

GIR profiles were smoothed by a locally weighted regression technique (LOESS, factor 0.10) using SAS software (version 9.4). The primary glucose-lowing effectiveness endpoints were the following parameters derived from the smoothed GIR profiles during the time interval from 0 to 10 h: (1) maximal GIR (GIR_max_), (2) AUC of GIR from 0 to 10 h (AUC_GIR, 0–10h_). The time-related parameters including the time of GIR_max_ (tGIR_max_), the onset of time (T_onset_) (defined as the first time when BG declining at least 0.28 mmol/L from baseline), the time for GIR to rise from 0 to half of GIR_max_ (T_early50%_), and the time for GIR to decline from GIR_max_ to half of GIR_max_ (T_late50%_) were also recorded. A similar analysis was performed for GIR_max_ and AUC_GIR, 0–10h_.

Time-related PK/PD parameters were evaluated by a nonparametric approach based on the Hodges-Lehmann method.

Considering a 20% within-subject variability in primary endpoint parameters of PK/PD following IAsp administration, and assuming a difference of 5% between test and reference, and a type-I error rate of 2.5%, a sample size of 30 subjects was planned (after considering 20% dropout rate) for the replicate crossover study to have 90% power to reject the null hypothesis that PK/PD of RD10046 was not bioequivalent to NovoRapid.

The safety assessments included the vital signs, physical examination, laboratory tests (complete blood cell count, liver and renal function, urinalysis, etc.), 12-lead ECG, injection site reaction, hypoglycemia, and other AEs.

### Ethics approval

Written informed consent will be obtained from each participant at the time of enrolling in the study. The trial has been approved by the Ethics Committee of West China Hospital of Sichuan University.

## Data Availability

The reasonable requests for data and materials should be addressed to Prof Yu.
