# Author Correction: Similar pharmacokinetics and pharmacodynamics of a new biosimilar and reference insulin aspart in healthy Chinese males

**DOI:** 10.1038/s41598-021-95661-9

**Published:** 2021-08-05

**Authors:** Hui Liu, Hongling Yu, Lisi Sun, Jingtao Qiao, Sainan Wan, Shuang Li, Jiaqi Li, Huiwen Tan, Yerong Yu

**Affiliations:** 1 Department of Endocrinology and Metabolism, West China Hospital, Sichuan University, No.37 Guoxue Alley, Wuhou District, Chengdu City, Sichuan Province People’s Republic of China; 2 Yichang HEC Changjiang Pharmaceutical Co., Ltd., No. 38 Binjiang Road, Yidu, Yichang City, Hubei Province People’s Republic of China

Correction to: *Scientific Reports* 10.1038/s41598-021-88782-8, published online 04 May 2021

The original version of this Article contained an error in the spelling of the author Sainan Wan which was incorrectly given as Sainan Wai. The original Article has been corrected.

